# Violent Radicalization and Post-traumatic Dissociation: Clinical Case of a Young Adolescent Girl Radicalized

**DOI:** 10.3389/fpsyt.2022.793291

**Published:** 2022-03-22

**Authors:** Julie Rolling, Guillaume Corduan, Martin Roth, Carmen M. Schroder, Amaury C. Mengin

**Affiliations:** ^1^Regional Center for Psychotraumatism Great East, Strasbourg University Hospital, Strasbourg, France; ^2^Department of Psychiatry, Mental Health and Addictology, Strasbourg University Hospital, Strasbourg, France; ^3^Centre National de la Recherche Scientifique Unité Propre de Recherche 3212 (CNRS UPR 3212), Institute for Cellular and Integrative Neurosciences (INCI), Strasbourg, France; ^4^VIRAGE Network (Violence of Ideas, Resources and Support in “Grand Est” Region), Maison des Adolescents, Strasbourg, France; ^5^Department of Psychiatry, Public Health Establishment of Northern Alsace, Brumath, France; ^6^Federation of Translational Medicine of Strasbourg, Strasbourg, France; ^7^INSERM U1114, Cognitive Neuropsychology and Pathophysiology of Schizophrenia, Strasbourg, France

**Keywords:** violent radicalization, complex psychotraumatism, post-traumatic stress disorder, post-traumatic dissociation, adolescence

## Abstract

**Introduction:**

Since 2014, the profiles of radicalized individuals have changed with the appearance of radical groups composed of a large proportion of adolescents. Various individual, relational, and social vulnerabilities have been identified as being involved in the radicalization process of adolescents. Among these factors, it appears that early and repeated history of personal and/or family psychotraumatism may constitute factors of vulnerability to violent radicalization.

**Material and Methods:**

The clinical situation of a 17-year-old woman convicted of “links with a terrorist group (DAECH)” was recruited from the 130 radicalized young people followed by the teams of the *Maison des Adolescents* and the Child and Adolescent Psychiatry Service of the University Hospitals of Strasbourg since May 2015. Based on the analysis of this clinical case, we present the hypothesis that post-traumatic antecedents can constitute vulnerability factors to violent radicalization, and that post-traumatic symptoms can be “used” by recruiters of radical movements at different moments of the radicalization process by reactivating post-traumatic psychic mechanisms, but also, for a smaller number of subjects, by the induction of the trauma (viewing of propaganda videos).

**Results:**

We show a possible link between violent radicalization and complex psycho-traumatism with an impact of the reactivation of post-traumatic mechanisms such as (i) the activation of the autonomic nervous system and emotional dysregulation on violent acts, (ii) the activation of dissociation mechanisms (psychic sideration and post-traumatic amnesia) on indoctrination and violent acts, (iii) the activation of control mechanisms on the search for a strict framework of life and a radical ideology and (iv) relational avoidance on the processes of relational rupture and radical socialization. Thus, we highlight that the radicalization process can respond to the needs and psychic functioning of psycho-traumatized individuals (channeling tensions, being recognized and active in one's life).

**Discussion:**

We discuss the central role of propaganda videos in the activation of the ANS and dissociation, and the self-perpetuating process between these two posttraumatic mechanisms. We also discuss clinical and therapeutic perspectives (therapies targeting complex psychotrauma).

**Conclusion:**

Psychotrauma can promote radicalization due to vulnerability mechanisms. Treatments targeting psychotrauma may be one of the ways to get these young people out of violent radicalization.

## Introduction

Since 2014, the profiles of radicalized individuals have evolved with the appearance of small radical groups composed of younger individuals with a significant proportion of radicalized adolescents (1, 2). The multiplication of radicalized youth profiles in France and Europe follows the creation of the “Islamic State” on 29 June 2014 in Syria. The development of new recruitment methods based on the dissemination of radical ideology via social networks has strongly contributed to the increase in recruitment among adolescents (1–5). In this context, since May 2015, our teams at the *Maison des Adolescents*^1^ (Violence of Ideas, Resources and Support in “Grand Est” region network, VIRAGE^2^ network) and the child and adolescent psychiatry department of the University Hospitals of Strasbourg have accompanied 130 adolescents and young adults who have joined violent radical movements (mostly Islamic). Violent radicalization is defined as “a process by which an individual adopts an extreme belief system -including the willingness to use, encourage or facilitate violence- in order to achieve the triumph of an ideology, a political project or a cause” (6). This process is the result of an encounter between an individual's background and an ideology generally promoted by a jihadist leader and/or group that is the source of recruitment and enlistment (propaganda, identification with the leader, radical socialization, etc.) (7).

### Radicalization Vulnerability Factors

The determinants of radical commitment are multiple (8) and include *individual, micro-environmental, relational and social vulnerability factors* combined with *international geopolitical factors* (7, 8). At the individual level, frequently described *psychological vulnerability factors* (9) are early experiences of abandonment (10), perceived injustice, experiences of stigmatization (7), personal uncertainty, dynamics of rebellion, violence and/or delinquency (3, 9), behavioral disorders (11) and depressive fragility (12, 13). Individual vulnerabilities are frequently associated with *micro-environmental and relational factors* such as family dysfunction, family breakdown and/or friendships with radicalized individuals (9). *Social factors* (level of education and professional integration) are also described (3, 14). There is therefore no single trajectory or linear causality leading to radicalization (15), but a risk of switching to radicalization at a period of psychological vulnerability (adolescence, loss, break-up) in individuals presenting a combination of different vulnerability factors.

Adolescence is a period of heightened vulnerability to radicalization (1, 10) due to the psychological changes (abandonment anxiety, autonomy from parents) and needs (search for identity, ideals and identification figures, search for group support) specific to this age of life (1, 16, 17). Oppositional behavior and extremist and violent positions can be a way to separate from parents and create one's own identity. In this context, integration into a radical group provides a sense of belonging that helps to combat anxieties of abandonment and soothes the adolescent (18). Moreover, adolescents are very receptive to Manichean and paranoid discourses because these discourses provide a clear vision of the world and propose a break with their parents' ideas. Violent radical groups are then very attractive cultures of opposition (19).

### Psychotrauma, Violence and Radicalization

Nevertheless, the specificities of adolescence are not sufficient to explain the attraction of adolescents to violent radicalization and other factors such as the traumatic childhood histories of these adolescents are increasingly appearing as vulnerability factors to be explored. Indeed, various teams working with radicalized adults and adolescents have identified a history of psycho-trauma in these populations (10). For example, Simi's work with 44 former members of violent white supremacist groups found that 45% reported having been physically abused as children and 21% reported having been sexually abused (11). With regard to young people involved in violent extremism, Simi's work suggests that these young people constitute a heterogeneous population of “ordinary offenders” with a history of exposure to violence (physical and/or sexual assault) (3, 10, 11). Concerning adolescents, Oppetit's work with 70 radicalized adolescents (81.4% female) shows 87.1% history of neglect or psychological abuse, 30.7% history of physical or sexual assault and 44.3% history of scarification in this population (8). Furthermore, among the 130 young people (67% female) followed since 2015 by the VIRAGE network, 55% have a post-traumatic history, 90% of which concerns repeated traumatic exposure (10). Single traumatic exposures appear to be less frequent (20). Other studies highlight trans-generational trauma (21), which illustrates the transversal aspect (individual and/or family) of the traumatic component in the radicalization process.

Furthermore, it is known that exposure to toxic stresses (adverse life events or psychotrauma) during childhood and adolescence impacts the neurodevelopment of the child emotionally, cognitively and behaviorally (22, 23). In the long term, the known consequences are variable (adjustment disorders, emotional and stress regulation disorders, behavioral disorders, aggression, etc.) (22–25) and frequently appear during vulnerable periods such as adolescence. *In situations* of early and repeated childhood trauma, different cognitive changes (changes in representations, self-perception, relationship to others) may participate in the appearance of aggressive behavior. Research on exposure to violence in 4,458 primary school children suggests that witnessing community violence influences children's aggressive behavior, both through imitation of violence and through the development of associated cognitions (aggressive fantasies and normative beliefs about aggression) (26). In total, numerous studies highlight the impact of early traumatic exposure on violent behavior (27, 28), particularly for complex psychotrauma (29), but also a link between psychotrauma in childhood and an increased risk of delinquency and between psychotrauma and radicalization.

Our working hypothesis is that personal and family post-traumatic antecedents may constitute vulnerability factors to violent radicalization (27, 28), and that post-traumatic symptoms may be “used” by recruiters of radical movements at different moments of the radicalization process by the reactivation of post-traumatic psychic mechanisms (in particular neurovegetative and dissociative), or for a more limited number of subjects, by trauma induction. We will illustrate our hypothesis by analyzing the clinical case of Lea (identity anonymized), recruited from among the 130 radicalized young people monitored by the VIRAGE network since May 2015. Regular psychiatric interviews (including the use of validated assessment scales) by two of the authors of the article were carried out over a period of 3 years and allowed us to collect all the data. Based on this clinical example, we will develop the hypothesis of a possible link between psychotrauma and violent radicalization, in particular concerning the impact of the reactivation of post-traumatic mechanisms such as (i) the activation of the autonomic nervous system (ANS), (ii) the activation of dissociative mechanisms (psychic sideration and post-traumatic amnesia), (iii) the activation of control mechanisms (search for a ritualized framework and radical ideology) and (iv) avoidance processes (relational avoidance and relational rupture). Thus, we will see how the radicalization process can respond to the needs and psychic functioning present in psycho-traumatized individuals.

Our analysis is based on a diagnostic approach by linking the different issues covered by the literature to Lea's experience and diagnosis.

## Lea, a Clinical Case Between Violence and Radical Commitment

Lea, 21, has been followed by the VIRAGE network team since 2017 following a conviction for “links with a terrorist group (DAECH),” “apology for terrorism” and “plans to leave for the Iraqi-Syrian zone.”

Lea was an only child. Her parents separated when she was 2 years old. Her mother remarried and retained primary custody. During this period, Lea was allegedly abused and tortured by her stepfather. The abuse is said to have started at the age of 7, when the stepfather became unemployed. Lea explained: “the first time he beat me, I didn't understand what was going on” and “I felt like I was no longer in reality.” From adolescence onwards, sexual violence and humiliation (“my stepfather looked at me naked in the shower”) were added. At this time, the adolescent attributes an omnipotence to her stepfather and an absence of protection from her mother (“my stepfather had always what he wanted,” “my mother saw everything and said nothing... she spent her time hating others”). It should be pointed highlighted traumas over three generations on the maternal side. In this context of daily violence, Lea went to live with her father at the age of 13. Her father was unaware of the physical and sexual abuse. A few months after moving in, her father died in a car accident under the influence of alcohol. Lea explained that she was in shock and that she “wished she could die to join her father.”

The return to her mother's house was associated with a sense of despair. Lea spoke of constant hyper-activation (feeling angry: “I was explosive”) combined with stomach aches. She explained that she could not sleep for fear that her stepfather would attack her again. Her sleep was interrupted by nightmares where she relived the attacks. Lea spoke of a deep sense of injustice and guilt that was constantly eating away at her (“I should have said no to my stepfather... I should have stopped my father from driving drunk”). She scarified herself daily to forget “the images that haunted her.” At school, Lea had few friends. She explains her situation by saying that the other children rejected her because of her sloppy clothes. At the time, Lea was empty and thought about committing suicide, but being Catholic, she was afraid of going to hell. She had imagined becoming a “nun” to go to “heaven,” but she was sexually harassed by a Catholic association official to whom she had confided her stepfather's violence.

Following this, Lea explained that she found help from a “Muslim spiritual guide” who was “like a father.” A friend introduced her to this man, explaining that he could help her feel better. This man was running an unofficial religious center. She had followed his precepts, which had calmed her down (“the prayers calmed me down, I didn't have stress attacks or scarifications anymore”). After a few months, while Lea was still a minor, she was introduced to a man who was to become her husband. The announcement of an imminent marriage precipitated the break-up with her mother, who threw her out of the house. Lea found herself isolated and under the influence of two men: her husband and her spiritual guide. A few months after her marriage, the “Muslim spiritual guide” began to sexually harass her. In response, her husband confined her to her home where she remained locked up for two years. She was criticized daily by her husband, whether it was about her religious practice, the way she looked after the house or her child.

It was in this context that Lea discovered fundamentalist preaching on the Internet. She experienced these preaching's as an escape and a way to feel valued (“it was the first time I could learn, that I wasn't put down”). Lea explained that when she watched the hateful preaching and propaganda videos of DAECH (images of beheadings) time stopped and feelings of fear subsided. These moments of “disconnection” encouraged Lea to continue watching these videos and to consider traveling to Syria to seek revenge and train to become a fighter in order to carry out a terrorist attack.

In total, at the time of her radicalization, Lea is a 17-year-old girl whose childhood was marked by early and repeated trauma such as neglect, physical and sexual abuse, humiliation combined with emotional deprivation and attachment disorders. Lea's trajectory is non-linear, with various attempts at self-regulation and self-dissociative behaviors (scarification, search for a frame of reference and an adult) preceding radicalization. The radical commitment was precipitated by the viewing of propaganda videos which led to his plan to leave for Syria.

Symptomatically, Lea presented with post-traumatic stress disorder, dissociative subtype (PTSD, defined by the DSM-5) associated with “extreme stress disorder not otherwise specified” (DESNOS, defined by the DSM-IV). The diagnosis of PTSD is confirmed by the Posttraumatic Stress Disorder Check List (PCL-5)^3^ (score of 52) with the presence of neurovegetative symptoms (hyperarousal, anger, internal tension, attention disorder, sleep disturbances with insomnia), relational avoidance, as well as mood disturbances (feelings of hopelessness) and negative cognitions (feelings of unfairness) (30). Dissociative^4^ items explored using the Peritraumatic Dissociation Experiences Questionnaire (PDEQ)^5^ indicate a recurrent and maximal dissociative state when watching violent videos or listening to indoctrination preaching (score of 43) (31). During these viewings, Lea describes states of bodily and temporal disconnection. Watching the videos dissociates Lea. The characterization of psychotrauma in DESNOS was confirmed by the Structured Interview for Disorder of Extreme Stress (SIDES)^6^ (32). In the adolescent, the symptoms of DESNOS are as follows:

A. *Impaired affect regulation and impulse control* with difficulty in emotional regulation, self-harm and anger.
B. *Impaired attention and awareness* with transient dissociative episodes, depersonalization and derealization and reliving.
C. *Altered self-perception* with feelings of helplessness, guilt and permanent harm.
D. *Alteration in interpersonal relationships*, whether in the perception of the aggressor (attribution of omnipotence to the aggressor) or in the relationship with others (isolation, withdrawal, rupture, search for a savior, failure to protect oneself).
E. *Somatization*.
F. *Alteration in belief and value systems* with a sense of hopelessness and loss of comforting beliefs.

PTSD is not always sufficient to define the complexity of clinical pictures in repeated trauma. In these case, the diagnosis of DESNOS and complex PTSD (defined by the International Classification of Diseases, 11th version, ICD-11) are mentioned (33, 34). In our work, we will therefore use by extension (and independently of ICD-11) the term complex psychotrauma to define the set of clinical symptoms of Lea. Thus, the various pathophysiological components of complex psychotrauma will be successively detailed to highlight their involvement in the radicalization process, such as:

- the activation of the autonomic nervous system (ANS) and of emotional dysregulation and impulse control during violent acts,
- the activation of post-traumatic dissociation (psychic and emotional sideration) and self-dissociative behaviors on indoctrination and violent acts,
- the activation of post-traumatic control mechanisms on the attraction of a strict life framework and radical ideology, in particular the link between:
  a. insecurity and alteration in the systems of meaning fostering adherence to the radical ideology,
  b. the alteration of self-perception and the instrumentalization of the sense of harm by recruiters,
- the alteration of relationships with others/relational avoidance on the processes of disruption, attraction to radical socialization and the search for a savior,
- and the impact of transgenerational trauma and a lack of identification on the appeal of the “jihadist ideal.”

## Psychotrauma and Radicalization

### The Impact of Autonomic Nervous System Activation, Emotional Dysregulation and Impulse Control

In Lea, as in other adolescents followed by VIRAGE, we can observe an alternating activation of two of the main post-traumatic mechanisms: activation of the autonomic nervous system and activation of dissociative mechanisms (see Section The Impact of Post-Traumatic Dissociation and Self-Dissociative Behavior). The activation of the ANS is responsible for the neurovegetative symptoms of PTSD, such as the hypervigilance, internal tension and anger present in Lea. These symptoms may be present either 'almost continuously' after a trauma, or they may be generated by exposure to sensory content related to a past trauma. Thus, Lea's state of internal tension (dysregulation of emotions and impulse control) diminishes her analytical and solution-finding abilities, which in turn diminishes her decision-making and may therefore facilitate indoctrination. In this context of physiological activation, the combination of internal tension with a sense of harm (see Section Alteration of Self-Perception and the Instrumentalization of Feelings of Prejudice by Recruiters), hatred or revenge can lead to violent acts (37). It should be remembered that lea was arrested just before the acts were committed. In addition, the hyperadrenergic activation state induces sleep disturbances such as insomnia and traumatic nightmares that can lead to a sleep debt (38) that sustains the amygdala hyperactivation.

Conversely, when dissociative mechanisms are activated, emotional anesthesia can induce a “desensitization to violence” responsible for an “attraction to violence.” In Lea, dissociation is observed during scarification or when watching propaganda videos (“I couldn't stop watching because the videos calmed me down, the images hypnotized me and time stopped, I was disconnected, I couldn't think anymore... it was like when I scarified myself”).

### The Impact of Post-traumatic Dissociation and Self-Dissociative Behavior

The link between dissociation and violent acts has been little studied, although dissociation is likely to precipitate violence since dissociative states create “an environment in which impulsive and violent acts are more likely to occur” (39, 40). Indeed, the association between psychotrauma and violence is even stronger when post-traumatic dissociative mechanisms are present (29), and the more severe the trauma, the higher the risk of a dissociative state (35, 41). For example, work on sexual abusers (42) has shown that traumatic exposure in childhood is associated with peri-traumatic dissociation at the time of abuse (31) and the development of a “dissociative personality trait” (35, 36). Moreover, dissociation is strongly associated with self-harming behaviors (scarification, suicide attempts) which are considered self-dissociating behaviors (43), and some authors have found a higher prevalence of self-harming behaviors in radicalized subjects (10, 36).

According to Vandevoorde, the dissociative state (feeling of unreality, loss of self-control, emotional anesthesia, loss of voluntary motor control, etc.) is a factor that may be involved in the initiation of aggressive motor behavior (44). However, the control of these behaviors is more complex in some adolescents due to a change in impulse control, classically theorized as the emergence of “violent and/or sexual impulses” (see Section The Impact of Autonomic Nervous System Activation, Emotional Dysregulation and Impulse Control). Therefore, the dissociative state may be sought by some young people like lea who seek to satisfy violent impulses or who need to soothe traumatic reactivations (45). Our hypothesis is that there is a strong association between experienced dissociation and acted dissociation in radicalized adolescents. This dissociative state would be sought by both the adolescents and the recruiters who would like to encourage a state of psychic numbness conducive to indoctrination and terrorist attacks. Furthermore, it is known that dissociative symptoms or a history of dissociative experiences could increase the risk of dissociation in situations of traumatic reactivation (exposure to elements related to a past aggression, exposure to one's own violence and/or exposure to traumatic scenes directly by recruiters). Thus, dissociation could be a facilitator of violent radicalization. In this context, the viewing of propaganda videos (images of fighting in the Iraqi zone) could contribute to reactivating post-traumatic dissociative mechanisms or induce a new trauma.

Currently, exposure to propaganda videos from social networks has become one of the most frequent means of indoctrination (4, 5). In these videos, content is designed to appeal to adolescents with youth-specific themes, such as camaraderie, adventure or the glorification and mystification of violence (4). These recruitment efforts allow membership to be presented as natural and 'fashionable'. Furthermore, the scripting is based on the juxtaposition of traumatic images and narcissistically empowering images. This scripting allows for an initial tolerance to the visual content offered, then when the individual is used to the images, the content becomes more violent, increasing the psychological shock. The use of music and subjective vision, frequently used in video games, also increases the dissociative effect of immersion and derealization (46).

For Lea, the substitution of scarification by the viewing of propaganda videos illustrates the search for self-dissociation, but also the potentially facilitating role of dissociation in radical engagement (reduction of internal tension, suspension of negative thoughts, absence of questioning, derealization, etc.). Indeed, the state of disconnection provoked by the videos facilitated radical engagement and the attraction for violence by Lea, who was looking for a way to “avenge the acts of her stepfather” (45). Moreover, the content of the videos allows for a glorification and narcissistic enhancement of her revenge.

However, the repetition of self-dissociative behaviors (scarification or “watching propaganda videos”) induces a chronic production of endorphins, which leads to a decrease in the brain's sensitivity to these hormones, thus increasing the recourse to these behaviors (Lea watches these videos for an average of 10 h a day). In the long term, the perpetuation of these behaviors leads to a state of psychological numbness with a decreased ability to reflect and self-regulate (see Section The Impact of Autonomic Nervous System Activation, Emotional Dysregulation and Impulse Control) (37). Psychological numbness is an essential element of indoctrination as it promotes adherence to the radical ideology (see Section Insecurity and Radical Ideology: Alteration in the Systems of Meaning Causing Adherence to the Radical Ideology and Submission to Its System of Thought), prevents questioning of the jihadist ideology and encourages acting out (a state of “automatic pilot”).

## Activation of Post-Traumatic Control Mechanisms and Attraction to a Strict Life Framework and Radical Ideology

### Strict Life Framework

The psychological disconnection induced by dissociation is reinforced by the recruiters of terrorist organizations, notably through the ritualization of daily life. On the other hand, the attraction of a strict living environment responds to Lea's and the radicalized youth's need to anchor themselves (3) and to their need for control. For Lea, this need for anchoring was illustrated by the search for a guide, first with a Christian association, then with the Islamist leader. We know that control mechanisms are frequent post-traumatic coping strategies that allow for a better understanding of the consequences of the trauma, particularly the insecurity linked to neurovegetative activation. In addition, lea and individuals who have been exposed to childhood trauma are also more insecure due to the frequent interaction with attachment difficulties (47), which further reinforces the need for a clear framework. In this context, the extreme ritualization of daily life (strict living environment with clothing or dietary requirements), the adoption of a radical ideology and indoctrination (need to learn many hadiths or to do frenetic reading) were important calming factors for Lea. Indeed, the physical (puberty) and psychic (abandonment anxiety and existential uncertainty) changes of adolescence strongly reactivated Lea's old traumas, in particular sexual traumas (1). Thus, for the adolescent, as for other young people, the change of clothing (wearing the hijab) made it possible to avoid the physical elements reminiscent of the aggression (post-traumatic avoidance symptoms) (17). This change also made it possible to codify the relationship with the body (post-traumatic control mechanisms), which also calmed Lea. The cessation of self-harm once Lea changed her lifestyle is diagnostic evidence of a decrease in her state of internal tension.

### Insecurity and Radical Ideology: Alteration in the Systems of Meaning Causing Adherence to the Radical Ideology and Submission to Its System of Thought

The association between insecurity, altered self-perception and loss of confidence in comforting beliefs presented by Lea is common in psychologically traumatized individuals. It has been shown that uncertainty, injustice and perceived threat are the three main cognitive determinants of adherence to a radical belief system (8, 48). Indeed, some research has found a correlation between perceived discrimination and engagement in radical behavior (9), as well as a link with traumatic experiences (48, 49). One hypothesis is that beliefs provide a sense of regaining lost control in traumatic contexts and reduce the stress experienced. The more uncertain an individual is in his or her environment, the more likely he or she is to adhere to a new belief and to identify massively with a group (13, 50). Thus, the adoption of a radical belief would provide security and “cognitive appeasement”.

### Alteration of Self-Perception and the Instrumentalization of Feelings of Prejudice by Recruiters

The different feelings (helplessness, guilt, lack of recognition and permanent prejudice) related to the altered self-perception were instrumentalized by Lea's recruiter. Thus, the recruiter used a paranoid discourse of victimization and rebellion that is particularly effective in victims (3). It is based on jihadist ideology which focuses on the “fault of the impure.” In this context, blaming others relieves the would-be jihadist of her own guilt inherited from previous trauma. In our clinical case, Lea felt guilty for the death of her father and “for letting her stepfather assault her”. Lea's guilt was displaced onto all adults. This is a radical logic of attribution from a personal problem. In this way, the recruiter can take advantage of post-traumatic vulnerabilities, creating compelling narratives that exploit the personal negative experiences of future recruits, distorting and misappropriating their beliefs (3). Among the psycho-traumatic antecedents, domestic violence is the situation most likely to induce a strong sense of injustice and guilt. Indeed, in the absence of a reassuring figure, a child as Lea is powerless. As she grew up, the feeling of injustice increases and fear turns into hatred, which is easily exploited by radical groups (6). In a context of power, hatred is displaced onto another individual, which will fuel the feeling of revenge and the will to take justice into one's own hands to regain control. In addition, the attribution of responsibility to the “unclean” also reinforces the perception of an external threat. This dehumanizes potential targets (“unclean”) and supports strategic plans to divide (“good guys and bad guys”) and eliminate the source of trauma (51). It creates a gap between the jihadist group and the rest of the world, which reinforces post-traumatic social avoidance mechanisms (see Section Post-Traumatic Relational Avoidance, Rupture and Radical Socialization).

## Post-Traumatic Relational Avoidance, Rupture and Radical Socialization

The alteration in Lea's interpersonal relationships (attribution of omnipotence to the aggressor, isolation, related with cognitive) is linked to cognitive changes and/or relational avoidance secondary to the trauma. Lea's relational avoidance (isolation at home, isolation at school) is a protective mechanism initially aimed at avoiding traumatic reactivation induced by contact with her abusive stepfather. This avoidance isolated the girl, which facilitated her recruitment by the jihadists.

Moreover, Lea's stepfather and mother had an authoritarian and terrorizing interpersonal styles frequently found in violent parents (14, 22). In these families, relational interactions are frequently marked by an insecure attachment style (47), alternating periods of fusion and/or rupture, as illustrated by Oppetit who shows 77% of abandonment experiences before the radicalization of these adolescents (10). These parents are unable to support their child's attempts at autonomy, because they consider them to be their own possession. As a reminder, when Lea announced her marriage, her mother directly cut off contact. The break-up is the only way out of this abusive environment and the feeling of powerlessness inherited from past abuse. Throughout the radicalization process, the dynamics of separation and break-up are actively reinforced by recruiters (8).

Cognitively, the thinking of these parents is characterized by an inaccessibility to doubt and a binary and Manichean view of the world, which is reflected in the interactions established with their children. Lea's parents tried to impose on their child a development strictly in line with their dysfunctional lifestyle. In this sense, their parenting style is similar to the parenting observed in some supremacist groups. Like Lea, children who have grown up in these environments are therefore later more likely to be receptive (insecurity, habits of a single, rigid discourse, etc.) to the influence of a charismatic, omnipotent leader and/or radical group. Recruitment will be facilitated by traumatic cognitive changes of attribution of omnipotence to the leader. On the other hand, recruitment by the group (social recruitment with the search for social inclusion and progressive integration) (52) will be favored by the search for a cohesive and reassuring group, which reduces the feeling of insecurity and the risk of relational instability secondary to psycho-trauma. Through the group, social interactions are mediated by social codes and a ritualization of life (see Section Strict Life Framework) that prevents overly threatening socialization to counteract avoidance. The radical group will also provide narcissistic and identificatory support. Finally, radical socialization^7^ also creates a “cognitive oligopoly” that reinforces jihadist belief as the radical ideology becomes dominant in the social reference group. The cognitive oligopoly reduces the sense of post-traumatic insecurity (see Session Insecurity and Radical Ideology: Alteration in the Systems of Meaning Causing Adherence to the Radical Ideology and Submission to Its System of Thought) (53).

## Transgenerational Trauma: The “Identification Gap” and Attraction to the “Jihadist Ideal”

The normalization of violence leading to violence against children is often the result of transgenerational trauma. In Lea's case, there was a repetition of psychotrauma over three generations (physical and sexual violence, war trauma). These traumas leave traces (transmission of the feeling of harm, post-traumatic amnesia). Thus, the transmission of the experience of harm from generation to generation can fuel the feeling of hatred (see Section Alteration of Self-Perception and the Instrumentalization of Feelings of Prejudice by Recruiters). Conversely, exposure to the traumatic story may be too painful, so members of the family system avoid talking about it and/or do not remember it (the dissociative mechanism of post-traumatic amnesia). Post-traumatic amnesia creates an apparent failure to transmit the traumatic story. Adolescents from these families find themselves without reference points or elements of identification for their identity construction (3).

Lea's search for a spiritual guide illustrates this phenomenon: “I didn't know anything about my family, so I needed something to hold onto.” In this context, the “jihadist ideal” of purity and perfection is a central element that will provide reference points and redefine the individual. This ideal will erase the feeling of post-traumatic powerlessness. This ideal is personified by a set of “identification figures” that define different radicalization profiles according to initial motivations (e.g., “ISIS as utopia myth,” “Mother Theresa myth,” “savior myth”) (45). These identificatory supports respond to the quest for identity and the search for status and importance which are strong motivations of psychotraumatized adolescents (8). They offer them the illusion of a grandiose identity and a break with their past as victims and their feeling of devaluation. Moreover, while the violence of psycho-traumatized adolescents is generally repressed by society, it is promoted and valorized through figures such as “the Lancelot myth”, which attracts and reassures young people.

## Impact of a Self-Sustaining Post-Traumatic Loop in Radicalization

**Figure 1:**
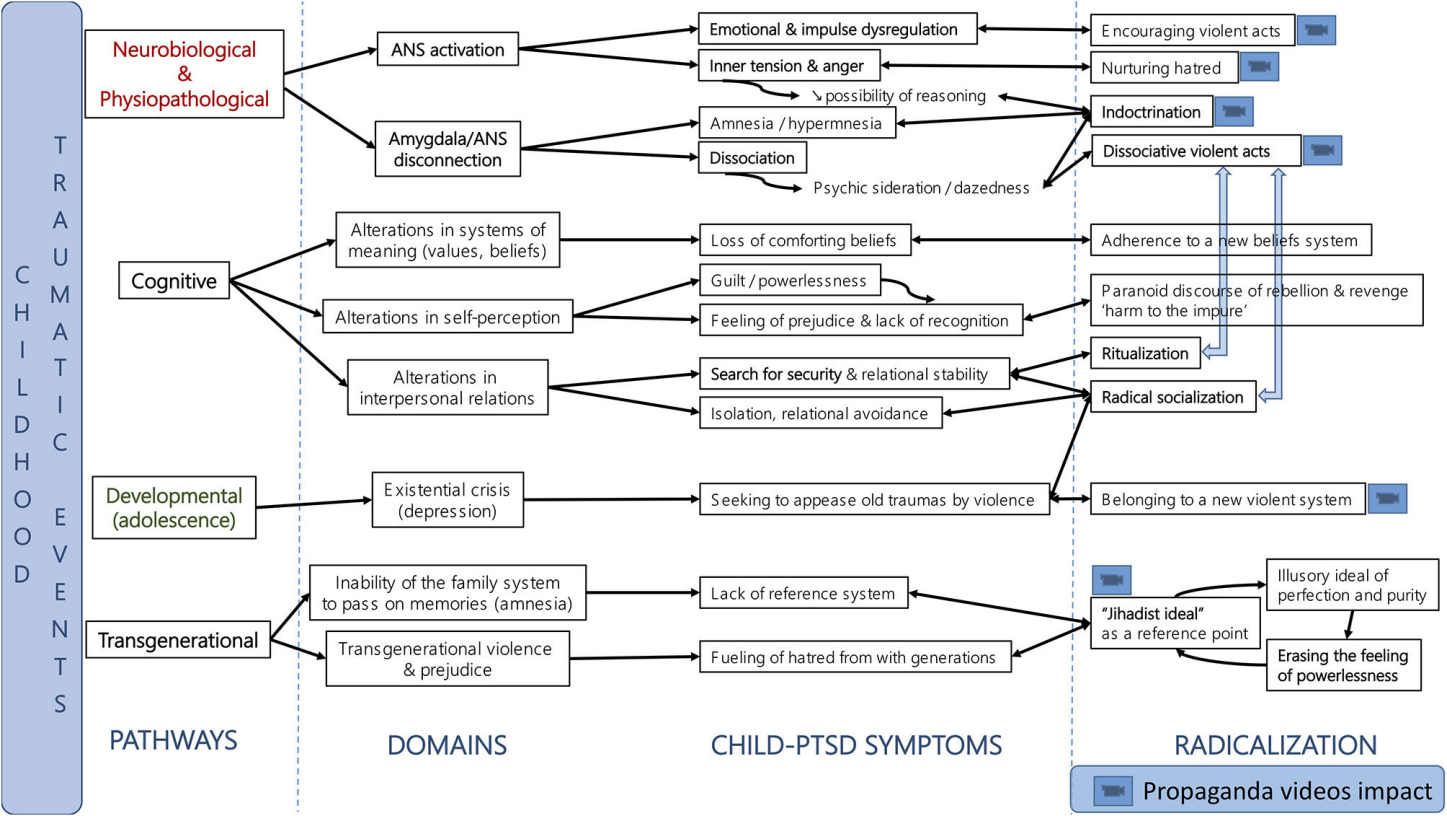
Summarizing the hypothesis of activation of post-traumatic mechanisms in radicalized adolescents.

The clinical case of Lea illustrates that a traumatic childhood past could therefore have an impact on the process of violent radicalization because of the victims' difficulty in channeling post-traumatic symptoms and the instrumentalization of these symptoms (see Figure 1). For example, Lea's recruitment was facilitated by the instrumentalization of insecurity (ritualization of daily life, provision of a reassuring and constraining ideology, leader dictating conduct), sense of harm (recognition of harm, promise of a new status, jihadist ideal), anger (valorization of the act) and relational avoidance (radical socialization, ritualization). Dissociation was essential in the indoctrination and the act. It was reinforced by the ritualization of daily life, adherence to a simplistic ideology and sleep deprivation. In this process of radicalization of Lea, the viewing of propaganda videos (violence, unreal atmosphere) has a central role of reactivation (reliving) and dissociation. On the one hand, the recruiters excite the adolescent (activation pathway ANS, neurovegetative activation, fear, hatred) and on the other hand they calm her (dissociative pathway, psychic sideration, emotional anesthesia, depersonalization) which traps the young person from her own post-traumatic mechanisms. The combination of activation and dissociation components creates a “hot”/“cold' that makes the adolescent dependent. In addition, the combination of emotional activation and psychic numbing promotes behavioral responses that decrease the adolescent's cognitive possibilities.

## Therapeutic Strategies

In Lea's case, the overall care was aimed at reducing neuro-vegetative activation and dissociation. A treatment plan incorporating element of trauma-focused therapies (dialectical behavior therapy for PTSD) was proposed to Lea (54). Thus, a first phase focused on securing Lea in order to increase her ability to tolerate and regulate her activation and dissociation states (54). This phase is based on the recognition of traumatic experiences and the regaining of self-control (emotional control, psycho-education and cognitive restructuring) (54). The second stage is guided exposure: it allows the dissociation to be treated by linking the physical, emotional and narrative components. During this stage, the therapist must avoid over-soliciting the neuro-vegetative system (e.g., stakes of success, confrontation with too strong emotional content). For Lea, psycho-boxing was chosen to create the situation of guided exposure. Psycho-boxing is a mind-body approach that allows to work directly on the relationship with violence (55). This technique uses free fighting with muffled strikes carried out in a safe environment. It is an empirical technique that has been used for 30 years in France with some violent prisoners. Our team was trained by the creator of this technique (55). In psycho-boxing, contact sports gestures are used to feel the emotions linked to violence and to externalize the internal tensions, feelings and representations associated with violence. This technique allows the reactivation of post-traumatic mechanisms and the control of this reactivation by the framework put in place (co-therapist, attenuated striking, immediate verbalization of the violence, etc).

Finally, it has been proposed that work on accepting the past be combined with a new life project centered on social relations (ability to create a solid relationship with a competent adult, ability to develop a sense of belonging to a group or an important cause) (56). For this last objective, Lea's family functioning and transgenerational trauma were assessed. Indeed, it is known that strong family support and exposure to the traumatic history between the child and his or her parents allows for an improvement in post-traumatic symptoms (57). Nevertheless, no family therapy (parent-child interaction interventions or multidimensional family therapy) could be proposed because there was a risk that it would be counterproductive (risk of instrumentalization of Lea's suffering by her family, reversal of responsibility on Lea).

## Discussion

Autonomic nervous system activation has often been described as a possible risk factor for radicalization (27, 28, 54). Recruiters use emotion-based recruitment strategies to increase ANS activation (54). Like other authors we show that the psychopathological processes of radicalization could involve “the continuous, deliberate and non-deliberate creation of trauma and toxic stress” (27). For some authors “highly stressful environments or environment-specific parenting or socialization techniques” are at the origin of toxic stresses (27, 28). Our hypothesis further highlights the key impact of the use of propaganda videos in activating the ANS and creating these negative stresses, but also in activating a post-traumatic self-regulation loop. Stress causes behavioral responses such as insecurity, increased aggression, impulsivity and other “quick life strategies” (27, 28). In this context, extremist involvement would be a coping strategy in difficult environments such as radicalization (11, 22).

In this perspective, Koehler postulates that the central mechanism of radicalization processes would be the combination of constant traumatic and therapeutic elements (e.g., the positive aspect of salvation, community, spirituality) to “form societies of contrast in which the individual could be psychologically trapped” (28). We highlight how the different factors potentially involved in radicalization (social support, belonging, belief, alternative identity) can be intrinsically modified by post-trauma mechanisms (see Figure 1) and instrumentalized by violent extremist groups. We therefore postulate that one way to counter radicalization processes is to decipher the post-traumatic component embedded in these risk factors. Furthermore, we postulate that the alternation between ANS arousal and dissociation keeps the individual in a state of psychological sideration, and that the management of this state (avoidance of activating situations, securitization, emotional control, etc.) is a prerequisite for all other therapies (especially therapies working on the cognitive components) and/or accompaniment.

### Strengths and Perspectives

One of the strengths of this article is that Lea's case is based on a clinical diagnosis and engagement with Lea in the context of a therapeutic intervention. Nevertheless, the explanatory value of most risk factors associated with entry into violent radicalization has been shown to be very low and should therefore be viewed with great caution (28, 55). Our study does not claim to generalize the links between trauma and radicalization, but aims to propose a framework for reflection and to raise awareness among clinicians and researchers of the need to detect trauma in radicalized adolescents. Thus, the vulnerabilities generated by psycho-trauma should prompt us to systematically explore post-traumatic histories in radicalized youth, even when another diagnosis has been made. Indeed, we know, for example, that depression is a comorbidity of PTSD. Cohen's team showed that 44% of them had suffered from depression before their radicalization (10). These deficits may increase vulnerability to recruitment by violent extremist groups as adherence to a radical ideology counteracts depressive symptoms (56). Nevertheless, it is complex to attribute depressive symptoms to isolated depression or a complication of PTSD, so screening for PTSD is always advisable.

In this respect, we should also be particularly vigilant with young people returning from the Iraqi-Syrian zone, whether or not they were previously radicalized. Indeed, these young people have frequently undergone repeated traumatic exposure (exposure to the atrocities of war, intimidation, violence) (3). Moreover, there is also a risk that they are traumatized by their own abuses. The prospect of an increase in the return of these young people from the Middle East to their country of origin should prompt an increase in existing mental health programs and support. The preponderance of post-traumatic vulnerabilities should also invite us to adapt the proposed intervention programs by favoring the integration of different efficient components (security, emotional control, psycho-education, etc.) in the therapies of complex psychotrauma (DBT-TSD, IRT, etc.). For example, elements of psycho-education can help to deconstruct radicalization pathways. The integration of psycho-corporal therapy also deserves to be considered. All of these therapeutic proposals remain exploratory but open up a field of possibilities in the field of psycho-traumatology.

### Limitations

Our work is based on a clinical case study, but the theoretical hypotheses formulated are also based on the observation of several similar cases and the link with the literature on the subject. They therefore have inherent limitations in this type of work, such as the difficulty of generalization, the risk of over-interpretation and the retrospective design. Thus, our hypotheses could be clarified by pooling the observations collected from several patients and by more systematic qualitative and quantitative evaluations. The neurobiological circuits or the impact of sleep evoked also remain hypothetical and based on our observations and could be confirmed by other studies.

## Conclusion

The importance of psychotrauma care in radicalization has been recognized by various bodies (Radicalisation Awareness Network, RAN, 2018) (57). In this light, the management of radicalized adolescents should include systematic clinical assessment and targeted psychotrauma care (DBT-TSD, IRT, etc.) and/or the integration of components of psychotrauma targeted therapies in specialized programs for radicalized and/or de-radicalized youth. It is also necessary to have a precise knowledge of the family (assessment of family structure and attachment), social, cultural and ideological context, in addition to the psycho-traumatic components. In this context, collaboration between services dealing with radicalized adolescents such as the VIRAGE network, educational and social services and child psychiatry and trauma and PTSD centers should be strengthened. This would allow for comprehensive work on the young person's mental health, trauma and environment. Thus, the training requirement for professionals involved in these situations concerns both the training of mental health professionals (prevention for teams taking care of young children), and teams involved with radicalized publics. Moreover, in a logic of prevention, a strong commitment from national and international decision-making bodies is necessary to support joint work between professionals involved in the follow-up of radicalized young people and the main actors of social networks. Finally, it is essential to continue research on the link between psycho-trauma and radicalization, by promoting the development of validated evaluation scales for this group. Furthermore, research evaluating the effectiveness of different therapies targeting complex trauma (DBT-PTSD, mentalization-based therapy, etc.) is also needed, in addition to the evaluation of specialized programs for radicalized and/or de-radicalized youth. Finally, the development of therapeutic tools using the digital format (avatar, video games) should be developed.

## Data Availability Statement

The raw data supporting the conclusions of this article will be made available by the authors, without undue reservation.

## Ethics Statement

This study on human participants was not subject to an ethical agreement because the Local Ethics Committee (University Hospital of Strasbourg) indicated that local legislation does not require the agreement of the Ethics Committee. Indeed, it is a matter of routine care and the clinical situation has been anonymzed. Furthermore, the clinical case was written when the subject was of age and fully capable of giving consent. Written informed consent was obtained from the individuals for the publication of any potentially identifiable images or data included in this article.

## Author Contributions

All authors listed have made a substantial, direct, and intellectual contribution to the work and approved it for publication.

## Conflict of Interest

The authors declare that the research was conducted in the absence of any commercial or financial relationships that could be construed as a potential conflict of interest.

## Publisher's Note

All claims expressed in this article are solely those of the authors and do not necessarily represent those of their affiliated organizations, or those of the publisher, the editors and the reviewers. Any product that may be evaluated in this article, or claim that may be made by its manufacturer, is not guaranteed or endorsed by the publisher.

## Footnotes

^1^*Maison Des Adolescents*: french reception and care structures for adolescents aged 11-25 and their families to provide a response to the suffering associated with adolescence.

^2^In 2017, the team of the *Maison Des Adolescents* of Strasbourg has been missioned by the Regional Health Agency to create the VIRAGE network (Violence of Ideas, Resources and Support in “Grand Est” region). The missions of the VIRAGE network are (i) to deploy primary prevention actions against radicalization, (ii) to offer support to people who have been radicalized or are at risk of radicalization and their families, (iii) to offer support and resources to the various professionals, and (iv) to organize networking.

^3^The PCL-5 is a 20-items self-report questionnaire that assesses PTSD symptoms according to DSM-5 criteria. This instrument allows for screening and reassessment of clinical status over time (30). A cut-off score of 38 suggests the presence of PTSD.

^4^Dissociation can occur at the time of the traumatic exposure; this is called peri-traumatic dissociation and lasts from a few hours to a few weeks after the event. In some situations, this state may become chronic and persist long after the trauma in various forms of “trait dissociation” or “structural dissociation” and/or be integrated into the clinical picture of PTSD with a dissociative subtype and/or constitute one of the symptoms of DESNOS.

^5^The PDEQ is a self-administered questionnaire assessing the presence and intensity of peritraumatic dissociation (31). A total score greater than or equal to 15 indicates the presence of significant peritraumatic dissociation for a given event.

^6^The SIDES was designed as a diagnostic and outcome measurement tool for patients with complex PTSD (32). Each response is scored between 0 and 3, with the presence of a pathological score (>1) for each category necessary to consider the total score significant.

^7^Radical socialization: constitution of a social group that is quickly composed only of radicalized individuals.
